# RNA-Seq Reveals the Angiogenesis Diversity between the Fetal and Adults Bone Mesenchyme Stem Cell

**DOI:** 10.1371/journal.pone.0149171

**Published:** 2016-02-22

**Authors:** Xin Zhao, Yingmin Han, Yu Liang, Chao Nie, Jian Wang

**Affiliations:** 1 BGI Education Center, University of Chinese Academy of Sciences, Shenzhen, Guangdong Province, 518083, China; 2 BGI-Shenzhen, Shenzhen, Guangdong Province, 518083, China; 3 Binhai Genomics Institute, BGI-Tianjin, Tianjin, Tianjin, 300308, China; 4 Geneis, Beijing, Beijing, 100094, China; Instituto Butantan, BRAZIL

## Abstract

In this research, we used RNA sequencing (RNA-seq) to analyze 23 single cell samples and 2 bulk cells sample from human adult bone mesenchyme stem cell line and human fetal bone mesenchyme stem cell line. The results from the research demonstrated that there were big differences between two cell lines. Adult bone mesenchyme stem cell lines showed a strong trend on the blood vessel differentiation and cell motion, 48/49 vascular related differential expressed genes showed higher expression in adult bone mesenchyme stem cell lines (Abmsc) than fetal bone mesenchyme stem cell lines (Fbmsc). 96/106 cell motion related genes showed the same tendency. Further analysis showed that genes like ANGPT1, VEGFA, FGF2, PDGFB and PDGFRA showed higher expression in Abmsc. This work showed cell heterogeneity between human adult bone mesenchyme stem cell line and human fetal bone mesenchyme stem cell line. Also the work may give an indication that Abmsc had a better potency than Fbmsc in the future vascular related application.

## Introduction

Mesenchymal stem cells (MSCs) are kind of self-renewal cells which are characterized by their multi-potential to differentiate into different cell types [1]. The criteria of International Society for Cellular Therapy (ISCT) defined that MSCs can differentiate into skeleton, cartilage, adipose and some other organs in vitro culture [2]. During the past decade, many researches showed that MSCs can facilitate the angiogenesis by secretion of pro-angiogenesis factors, such as VEGF and HGF. The factors that mentioned could contribute to cardiac repair and enhance the reparative progress [3–4]. Also, majority MSCs would die after injected in vivo in the ischemic conditions [5]. With this unique character, many researches applied to MSCs in their survival and pro-angiogenesis capacity *in vivo* and *in vitro* research [6]. Some researchers showed that during hypoxia condition, MSC regulated myocardial angiogenesis by increasing the expression of VEGF [7]. However, it is still hard to distinguish which one was better in facilitating new blood vessel growth between Abmsc and Fbmsc.

Gene expression levels can differ widely between similar cells. Pioneering work in transcriptional profiling has been carried out by either multiplex quantitative PCR (qPCR) or microarray technology aiming to distinguish different MSCs [8]. Recently the maturation of single cell RNA-seq techniques made it easier to describe the expression profile at single cell and single base resolution [9–10]. With its particular transcript factors and target gene, each cell reveals a unique expression state. In this study, we focused on the difference between the single Abmsc and Fbmsc, hope to find the differences during the limited samples.

## Material and Methods

### Cell culture

Adults BMSCs single cells (HUXMA-01001, from 18–45 years old donors, Abmsc) and Fetal BMSCs single cells (HUXMF-01001, from aborted donors, Fbmsc) were obtained from Cyagen Biosciences Inc. (Guangzhou, China). The culture medium contained mesenchymal stem cell growth medium (Cyagen Biosciences Inc, Guangzhou, China) supplemented with 15% fetal bovine serum (FBS) (Hyclone), 100 IU/ml penicillin (Hyclone) and 100 mg/ml streptomycin (Hyclone). Medium was changed every 2–3 days. With the same culture conditions in other research, 5 samples of single hESC cells were downloaded from GSE 36552.

### Sample preparation

The step-by-step RNA-Seq method was described by Tang, et al [11]. Briefly, we used a mouth pipette to pick cells manually and transfered it into lysate buffer. We used the microscope to ensure only one cell was in the pipette. Then we started to perform the reverse transcription reaction on the whole-cell lysate. We used terminal deoxynucleotidyl transferase to add a poly (A) tail to the 3’end of the cDNA, and then performed 24+9 cycles of PCR to amplify the single cell cDNA. The bulk cell sample was followed the same procedure with about 10000 cells after MSCs were cultured.

### Real-time PCR

The quality of single cell DNA was analyzed by real-time PCR (TaqMan). We used qPCR analysis of a set of house-keeping genes to check the quality of the amplified single cell DNA (The primers of TaqMan were in the S1 Table and the normal qPCR primers were got with the Human Housekeeping Gene Primer Set (Takara, China)). The TaqMan procedure was performed using an ABI Step One with 96-well plates as follows: first, 50°C for 2 min to pre-heat the mixture, then 95°C for 10 min to activate the Taq polymerase, finally, 40 cycles of 95°C for 15 s and 60°C for 1 min. only the expression of the house-keeping genes in the same level were chose for the next RNA-seq.

### RNA-Seq library preparation, sequencing and alignment

After the harvest of DNA from a single cell, 100 ng of DNA was sheared into 250bp fragments by BiorupterTM Pico (Diagenode, Belgium). The library preparation was followed by BGI’s standard procedure. Shortly, the fragment was end-repaired, dA-tailed, adaptor ligated and then with a 4-cycle PCR program. The libraries were sequenced on the Illumina HiSeq 2500 platform using the 50-bp pair-end sequencing strategy. With Aaron M Streets ‘s single cell sequence advice [12], we get 11 samples with 75M reads and 14 samples with 6M reads, and a total of 44.012 GB of data(raw data) was obtained for all the samples together. We subsequently used samples with Q20 > 90% and Q30 > 85% for further analysis. In total, 25 sample cells within the RNA-Seq data set met all of these criteria for the final analysis. The hg19 RefSeq (RNA sequences, GRCh37) was downloaded from the UCSC Genome Browser (http://genome.ucsc.edu). We used the Tophat (v.2.0.12) [13] to align the filtered reads to the hg19 RefSeq. Finally, from these 44.012 GB of filtered reads, 34.1 GB (77.3%) of data were mapped to the hg19 reference databases.

### Identify of differentially expressed (DE) genes

To analyze differences in gene expression among the three different kinds of stem cells, we used Cufflinks (v.2.2.1) [14] to assemble and compare transcripts. Gene expression was calculated using the FPKM method (Fragments Per Kilobase transcriptome per million reads) [15]. We used all of the genes with FPKM≥1 as the expressed genes in the following analysis. During the procedure, P value (two-tailed) was calculated accordingly (two-sample t-test). Corrections for false-positive (type I errors) was performed using Benjamin’s false discovery rate (FDR) [16]. We use “P<0.05, FC (fold change)>2 or <0.5 and FDR<0.05” as the threshold to judge the significance of gene expression differences between Abmsc and Fbmsc. The data set was normalized by the SPSS 20 (SPSS, Inc., Chicago, IL, USA). The heatmaps were drawn by using the R packages as follows: function ‘pheatmap’, ‘gplots’ and ‘preprocessCore’ package, with complete distance and hierarchical clustering method.

### Reconstruction of co-expression network

This study performed WGCNA analysis to construct the modules of co-expression gene for the stem cell associated networks and their interactions. From the processed expression files, the networks were formed from the weighted correlation matrices following the protocols of WGCNA. Briefly, the WGCNA converts the gene expression profiles into connection weights that can be visualized as topology overlap measures (TOM). We defined modules using a hierarchical cluster method, and used the topological overlap dissimilarity measure (1-TOM) as the distance measure with a height cutoff value of 0.9 and a minimum size (gene groups) cutoff value of 10 for the resulting dendrogram. All network analyses were implemented in the package ‘WGCNA’ and ‘flashClust’ in the R environment as Li A described [17].

### Gene Ontology (GO) analysis

GO enrichment was performed using DAVID (http://david.abcc.ncifcrf.gov/) or Gene ontology (http://geneontology.org/) [18]. A hypergeometric test with the Benjamin and Hochberg false discovery rate (FDR) was performed using the default parameters to adjust the P value. The function ‘ClueGo’ in the Cytoscape v3.2 was designed for integrating heterogeneous expression data and functional network information [19]. So we used it to pick the hub genes and modules in the Cytoscape environment with the parameters of “P<10^−5^, %associated gene > 8 and kappa = 0.5”.

## Results

### Generation of RNA-seq

We generated 44 GB raw data from 8 Abmsc, 15 Fbmsc, one Adult bmsc bulk cell and one Fetal bmsc bulk cell. The generation of data from hESC was downloaded from NCBI database. All samples were separated into two parts for sequencing; the first part of the sample was on average 75 million 100-bases long reads, while the second part of the sample was on average 6 million 100-bases long reads (S2 Table).

### Transcriptional profiles across different cell types

**Fig 1:**
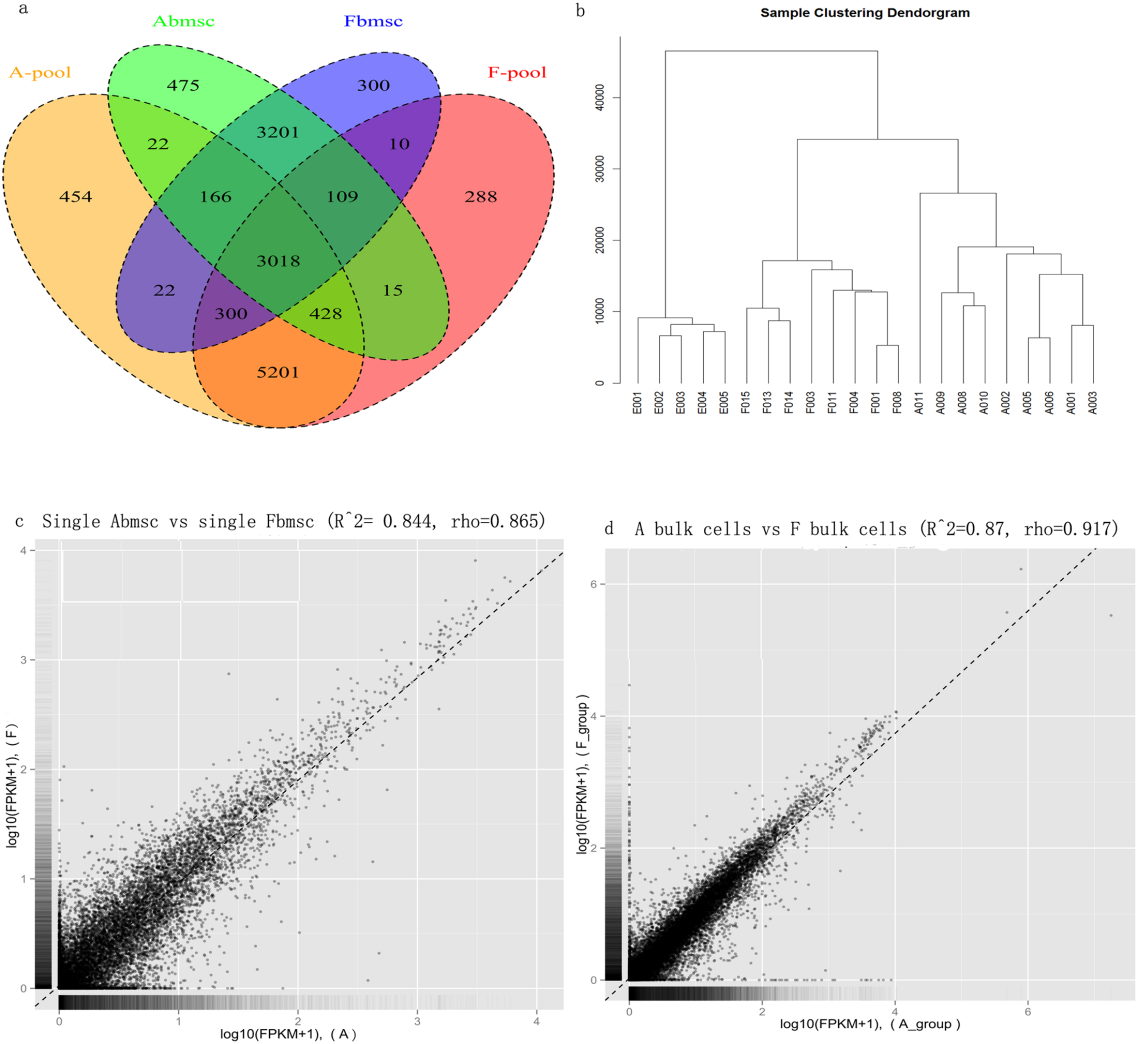
The general results of the RNAseq. a) The distribution of genes in single cell sample and bulk cell sample with the FPKM≥1, red: Fetal bmsc bulk cell, blue: Fetal bmsc, green: Adult bmsc, orange: Adult bmsc bulk cell; b-e) The Pearson and Spearman correlation coefficient were calculated between different groups. b) Abmsc and hESC; c) Abmsc and Fbmsc, d) Fbmsc and hESC; e) Adult bmsc bulk cell and Fetal bmsc bulk cell; e) Unsupervised clustering of the transcriptome of single cell sample. All RefSeq genes expressed in at least one of the samples with FPKM ≥ 1 were used for the analysis, with the order of hESC, Fbmsc and Abmsc.

We first analyzed how many known genes were expressed in each of the 25 samples. On average, we detected the expression of Abmsc, Fbmsc, Abmsc bulk cell and Fbmsc bulk cell with the number of 7434(31.9%), 7126(30.6%), 9611(41.2%) and 9369(40.2%) when compared to 23289 RefSeq genes(Fig 1a).

**Fig 2:**
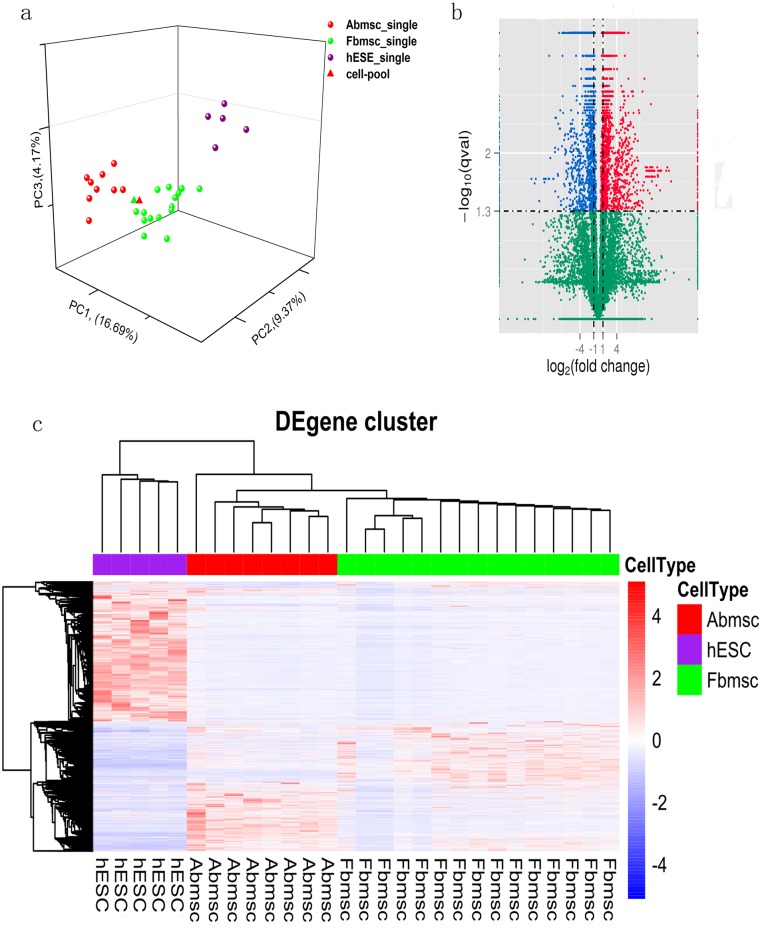
The DEgenes among the three cell lines , a) Principal-component analysis (PCA) of the transcriptome of three different cells. The different color circles indicated the three different cell lines. PCA1, PCA2 and PCA3 represent the top three dimensions of the genes showing differential expression among these cells, which accounts for 16.69%, 9.37% and 4.17% of the expressed RefSeq genes, respectively. b) Volcano plot of genes differentially expressed in Abmsc and Fbmsc samples. The log2 fold change difference between the samples was represented on the x-axis, and negative log of q-values is represented on the y-axis. Each point represents one gene, which had detectable expression in both samples. The genes differentially expressed in Abmsc compared with Fbmsc were plotted in blue for down-regulated genes and red for up-regulated genes, and non-significant genes are shown as green points. c) Clusters of genes showing representative expression patterns during three cell lines. We selected all of the genes that were differentially expressed between any two samples (fold change > 2 or <0.5, P < 0.01).

To determine whether these gene expression profiles have different correlations among cell lines, we analyzed RNA-seq data of Abmsc, Fbmsc and hESCs by Pearson correlation coefficient and unsupervised hierarchical clustering (Fig 1b–1d, S1 Fig). The result showed that Abmsc and Fbmsc bulk cell get closer distance than single cells, hESCs were far from both MSCs. Similar cell expression was also supported by principal-component analysis (Fig 2a). Next, we started to analysis the differential expression genes (DEgenes) between the three different cell groups. With the threshold of “P<0.05, FC (fold change)>2 or <0.5, FDR<0.05 and the union set among three groups” 4948 genes were judged as the DEgenes. During these genes, 948 genes showed up-regulated and 768 genes showed down-regulated when compared between Abmsc and Fbmsc, other genes showed significance when compared with hESCs (Fig 2b and 2c, S1 File). We carried out GO terms analysis for these differentially expressed genes and found the up-regulated genes were enriched for GO terms related to Extracellular matrix organization(Enrichment Score: 9.11, p = 4.3×10^−12^), Cell motility (Enrichment Score: 7.14, p = 3.4×10^−9^) and Angiogenesis (Enrichment Score: 7.03, p = 8.4×10^−9^); down-regulated genes were only enriched for Cell cycle related terms (Enrichment Score: 11.2, p = 8.3×10^−11^).

Interestingly, when we compared the 2 different bulk cell samples, we could only find 48 DEgenes in the comparison (17 up-regulated genes and 31 down-regulated genes, data no shown). This was also supported by the relatively short distance between these two groups of bulk cell in the PCA analysis.

### Reconstruction of network and evaluation of module significance by gene ontology term enrichment

In the first analysis, we identified 4948 genes as DEgenes that have significant expression difference among the three different kinds of cells. These DEgenes were used to reconstruct the co-expression network and identify a number of modules of high co-expression genes with the help of WGCNA. We shifted the module with the threshold of gene significance >0.4 and P value <0.01, and found that 6 modules matched (S2 Fig, S3 Table). Next we used the ClueGo in the Cytoscape (v3.2.0) to analysis the relationship between pathways and the six modules, with the parameters of “P<10^−5^, %associated gene > 6 and kappa = 0.5”. Only two modules were enriched in GO terms related to specific Biological Processes (BP): module blue and module red.

**Fig 3:**
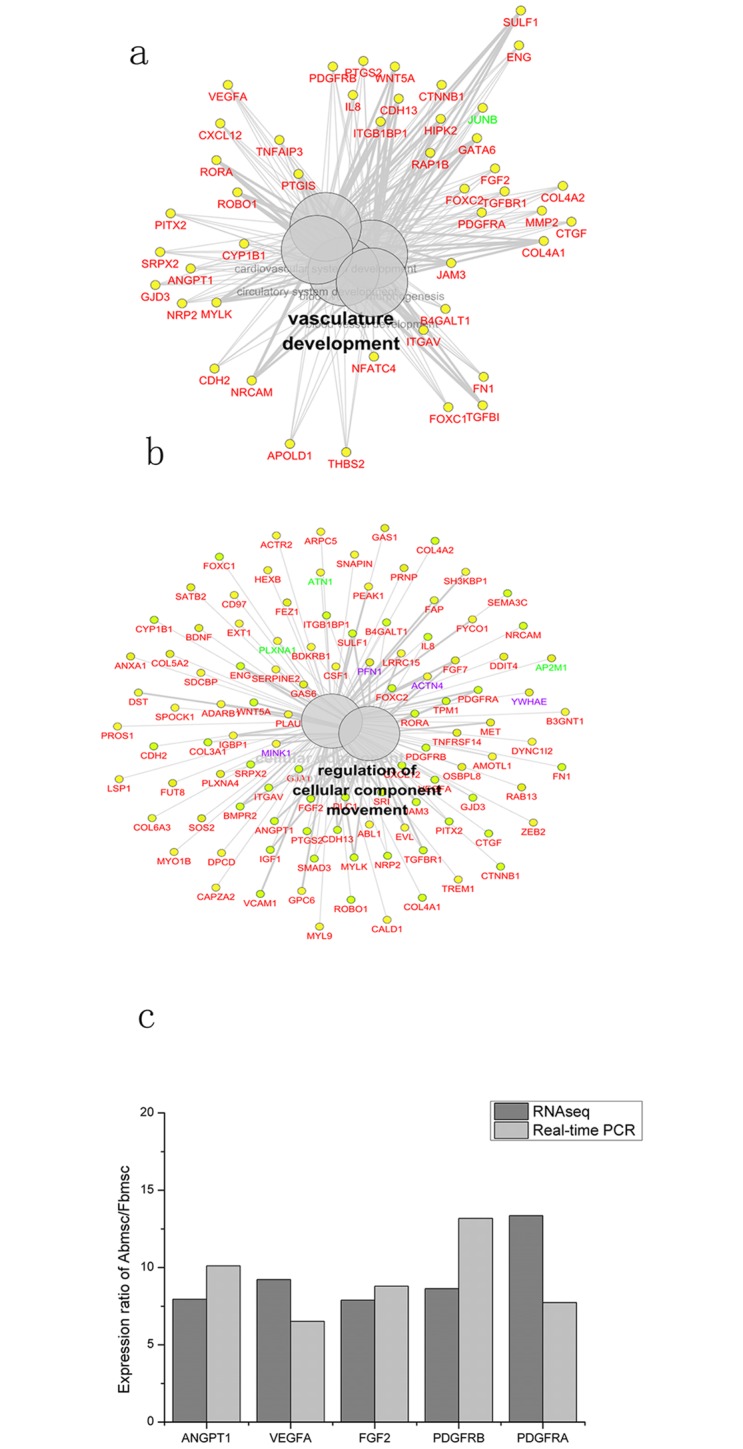
WGCNA analysis on the DEgene datasets. a,b) With the function of ClueGo in Cytoscape, graphic depiction of blue module (the vasculature development, regulation of cellular component movement) was shown. Before each viewing modules, parameters of P<10^−5^, %associated gene >8, and kappa = 0.5 were used to decrease the genes and Go terms in the module. The colors in the network showed the different cell lines expression. Red mean Abmsc showed the highest expression among the cell lines; while green mean Fbmsc and purple mean hESC. c) The relative expression of ANGPT1, VEGFA, FGF2, PDGFB and PDGFRA between Abmsc and Fbmsc.

Module blue consisted of 5 clusters, including: vasculature development, regulation of migration, cellular response to chemical stimulus, negative regulation of cellular process and extracellular matrix organization, other genes were focused on the abolished terms. During the five GO term groups, the top 5 significant GO terms were blood vessel morphogenesis (GO: 0048514, %associated gene = 9.12, P-value = 3.13×10^−9^), vasculature development (GO: 0001944, %associated gene = 8.82, P-value = 5.67×10^−10^), blood vessel development (GO: 0001568, %associated gene = 8.70, P-value = 2.95×10^−09^), regulation of cellular component movement (GO: 0071495, %associated gene = 8.58, P-value = 1.54×10^−09^) and movement of cell or subcellular component (GO: 0006928, %associated gene = 6.25, P-value = 9.7×10^−09^). Based on the significance of GO terms, we focused the ‘vasculature development group’ and ‘regulation of migration group’ for the detailed analysis. Vasculature related development showed that 44/45 genes had higher expression in Abmsc than Fbmsc (Fig 3a). Meanwhile, Cell motion showed that during the 104 DE genes, 96 genes showed higher expression in the Abmsc than Fbmsc (Fig 3b).

*ANGPT1*, *VEGFA*, *FGF2*, *PDGFRB* and *PDGFRA* were 5 genes that showed the significant fold changes (FC>6) in the blue module (Fig 3c), besides, these genes had a strong relationship with angiogenesis and cell motion. Former researches showed that *VEGFA* is a key regulator in vascular development and the progress of vascular development may involve a lot of effectors, including ERKs, PI3K/Akt, FAK and MAPK [20]. Also, tumor angiogenesis can result in a dysfunctional vasculature relied on *VEGFA* and its effectors [21]. So in order to decrease tumor angiogenesis and its motion, many researches tried different method to inhibit the expression of *VEGFA* [22–23]. Angiopoietins (*ANGPT1*) are proteins with important roles in vascular development and angiogenesis. Some research showed that by inhibiting the expression of *ANGPT1*, the integrity of the vascular will be decreased [24]. Others showed that in some disease model like acute pancreatitis, the increase of the *ANGPT1* could reduce the symptom by increasing the angiogenesis [25]. *FGF2* is one member of fibroblast growth factor family, which had a close relationship to tumor development. Some researchers found that during the hypoxia environment, tumor development became easier. And this progress increased the angiogenesis by *FGF2* [26]. Decreasing the expression of FGF2/EGR-1 pathway may also become an effective method to reduce the angiogenesis and vascular motion in small cell lung cancer [27]. *PDGFRB* and *PDGFRA* are two members of platelet-derived growth factor family, the expression of both genes could also induce angiogenesis by increasing the number of platelet [28–29].

Module red showed only one cluster: Golgi vesicle transport; other genes were focused on the abolished terms. The top significant GO terms were single-organism membrane budding (GO: 1902591, %associated gene = 30.30, P-value = 7.98×10^−10^), and vesicle targeting, to, from or within Golgi (GO: 0048199, %associated gene = 29.03, P-value = 8.78×10^−09^). Based on the GO term research, we focused on the ‘Golgi vesicle transport group’ for the further analysis. 29/30 genes were found to have higher expression in Fbmsc than Abmsc (S3 Fig). However, no significant relationship was found between Golgi transport and selected genes.

## Discussion

This study used single cell sample to have the transcriptome sequencing. The samples were from Abmsc and Fbmsc. These results provide a framework of 23 single cell samples and 2 bulk cell samples. To our knowledge, this is the first time that distinguishes two MSC cell lines expressing profile from single cell level. During this research, we found an interesting phenomenon. The samples from Adult and fetal MSCs showed significant differences in the single cell expression level. However, these results were not significant between bulk cell samples. The results showed that just like other single cell researches, MSCs may also had heterogeneity in single cell level [30–31]. That’s why bulk cell may have less DE genes than single cell samples.

It is hard for us to explain why Abmsc had a higher expression in angiogenesis than Fbmsc. We may prove it in the further research. However, we think it may have some relationship with the age that the donors hold. The age of 20–30 expressed the “gold” of a life. People will get the highest bone mass, the maximum metabolism level during the whole life. Therefore, the metabolism of MSCs may stand a high level. With the higher metabolism, the angiogenesis will become faster and stronger.

Because the cell lines were from the liquid nitrogen cryopreservation rather than the fresh tissue, the cell debris made the selection more arduous, besides, it was hard to get enough samples for test at the same time, the results may not as perfect as we designed. Nonetheless, our results lead the way for dissecting the molecular regulation of different mesenchymal cells and the developmental potentiality between Abmsc and Fbmsc.

## Supporting Information

S1 Fig The Pearson and Spearman correlation coefficient were calculated between different groups. a) single Abmsc vs single hESC; b)single Fbmsc vs single hESC. All RefSeq genes expressed in at least one of the samples with FPKM ≥ 1 were used for the analysis.
(TIF)

S2 Fig The Cluster Dendrogram and Module Significance, dendrogram showed relationship for the topological overlap of genes and their relationship to modules, which are color-coded. The picked module was matched two requirements: a) the cluster with the minim gene cluster of 10 and b) with the higher gene significance of 0.4. Other modules were abolished.
(TIF)

S3 Fig WGCNA analysis on the DEgene datasets, With the function of ClueGo in Cytoscape, graphic depiction of red module (Golgi vesicle transport) was shown. Before each viewing modules, parameters of P<10^−5^, %associated gene >8, and kappa = 0.5 were used to decrease the genes and Go terms in the module. The colors in the network showed the different cell lines expression. Red mean Abmsc showed the highest expression among the cell lines; while green mean Fbmsc and purple mean hESC.
(TIF)

S1 File the expression of DE genes. (XLS)

S1 Table The primer of the TaqMan. (DOCX)

S2 Table Basic quality of RNA-seq. (DOCX)

S3 Table The P value of the module in the network. (DOCX)

## References

[1] De Kock J, Najar M, Bolleyn J, Al Battah F, Rodrigues RM, Buyl K, et al Mesoderm-derived stem cells: the link between the transcriptome and their differentiation potential. Stem Cells Dev. 2012; 21(18):3309–23. 10.1089/scd.2011.0723 22651824

[2] Strioga M, Viswanathan S, Darinskas A, Slaby O, Michalek J. Same or not the same? Comparison of adipose tissue-derived versus bone marrow-derived mesenchymal stem and stromal cells. Stem Cells Dev. 2012; 21(14):2724–52. 10.1089/scd.2011.0722 22468918

[3] Copland IB. Mesenchymal stromal cells for cardiovascular disease. J Cardiovasc Dis Res. 2011; 2(1):3–13. 10.4103/0975-3583.78581 21716750 PMC3120270

[4] Huang W, Zhang D, Millard RW, Wang T, Zhao T, Fan GC, et al Gene manipulated peritoneal cell patch repairs infarcted myocardium. J Mol Cell Cardiol. 2010; 48(4):702–12. 10.1016/j.yjmcc.2009.10.032 19913551 PMC2905838

[5] Zhang D, Fan GC, Zhou X, Zhao T, Pasha Z, Xu M, et al Over-expression of CXCR4 on mesenchymal stem cells augments myoangiogenesis in the infarcted myocardium. J Mol Cell Cardiol. 2008; 44(2):281–92. 10.1016/j.yjmcc.2007.11.010 18201717 PMC2601571

[6] Aicher A, Brenner W, Zuhayra M, Badorff C, Massoudi S, Assmus B, et al Assessment of the tissue distribution of transplanted human endothelial progenitor cells by radioactive labeling. Circulation. 2003; 107(16):2134–9. 12695305 10.1161/01.CIR.0000062649.63838.C9

[7] Samper E, Diez-Juan A, Montero JA, Sepulveda P. Cardiac cell therapy: boosting mesenchymal stem cells effects. Stem Cell Rev. 2013; 9(3):266–80. 10.1007/s12015-012-9353-z 22350458

[8] Kern S, Eichler H, Stoeve J, Kluter H, Bieback K. Comparative analysis of mesenchymal stem cells from bone marrow, umbilical cord blood, or adipose tissue. Stem Cells. 2006; 24(5):1294–301. 16410387 10.1634/stemcells.2005-0342

[9] Ramskold D, Luo S, Wang YC, Li R, Deng Q, Faridani OR, et al Full-length mRNA-Seq from single-cell levels of RNA and individual circulating tumor cells. Nat Biotechnol. 2012 8; 30(8):777–82. 22820318 10.1038/nbt.2282 PMC3467340

[10] Tang F, Lao K, Surani MA. Development and applications of single-cell transcriptome analysis. Nat Methods. 2011;8(4 Suppl):S6–11. 10.1038/nmeth.1557 21451510 PMC3408593

[11] Tang F, Barbacioru C, Nordman E, Li B, Xu N, Bashkirov VI, et al RNA-Seq analysis to capture the transcriptome landscape of a single cell. Nat Protoc. 2010; 5(3):516–35. 10.1038/nprot.2009.236 20203668 PMC3847604

[12] Streets AM, Huang Y. How deep is enough in single-cell RNA-seq? Nat Biotechnol. 2014; 32(10):1005–6. 10.1038/nbt.3039 25299920

[13] Kim D, Pertea G, Trapnell C, Pimentel H, Kelley R, Salzberg SL. TopHat2: accurate alignment of transcriptomes in the presence of insertions, deletions and gene fusions. Genome Biol. 2013; 14(4):R36 23618408 10.1186/gb-2013-14-4-r36 PMC4053844

[14] Trapnell C, Roberts A, Goff L, Pertea G, Kim D, Kelley DR, et al Differential gene and transcript expression analysis of RNA-seq experiments with TopHat and Cufflinks. Nat Protoc. 2012; 7(3):562–78. 10.1038/nprot.2012.016 22383036 PMC3334321

[15] Trapnell C, Williams BA, Pertea G, Mortazavi A, Kwan G, van Baren MJ, et al Transcript assembly and quantification by RNA-Seq reveals unannotated transcripts and isoform switching during cell differentiation. Nat Biotechnol. 2010; 28(5):511–5. 10.1038/nbt.1621 20436464 PMC3146043

[16] van den Oord EJ. Controlling false discoveries in genetic studies. Am J Med Genet B Neuropsychiatr Genet. 2008; 147B (5):637–44. 18092307 10.1002/ajmg.b.30650

[17] Li A, Horvath S. Network neighborhood analysis with the multi-node topological overlap measure. Bioinformatics. 2007; 23(2):222–31. 17110366 10.1093/bioinformatics/btl581

[18] Huang da W, Sherman BT, Lempicki RA. Systematic and integrative analysis of large gene lists using DAVID bioinformatics resources. Nat Protoc. 2009; 4 (1):44–57. 10.1038/nprot.2008.211 19131956

[19] Bindea G, Mlecnik B, Hackl H, Charoentong P, Tosolini M, Kirilovsky A, et al ClueGO: a Cytoscape plug-in to decipher functionally grouped gene ontology and pathway annotation networks. Bioinformatics. 2009; 25(8):1091–3. 10.1093/bioinformatics/btp101 19237447 PMC2666812

[20] Ferrara N. Vascular endothelial growth factor: basic science and clinical progress. Endocr Rev. 2004; 25(4):581–611. 15294883 10.1210/er.2003-0027

[21] Claesson-Welsh L, Welsh M. VEGFA and tumour angiogenesis. J Intern Med. 2013; 273(2):114–27. 10.1111/joim.12019 23216836

[22] Chai ZT, Kong J, Zhu XD, Zhang YY, Lu L, Zhou JM, et al MicroRNA-26a inhibits angiogenesis by down-regulating VEGFA through the PIK3C2alpha/Akt/HIF-1alpha pathway in hepatocellular carcinoma. PloS one. 2013; 8(10):e77957 10.1371/journal.pone.0077957 24194905 PMC3806796

[23] Shen K, Ji L, Lu B, Xu C, Gong C, Morahan G, et al Andrographolide inhibits tumor angiogenesis via blocking VEGFA/VEGFR2-MAPKs signaling cascade. Chem Biol Interact. 2014; 218:99–106. 10.1016/j.cbi.2014.04.020 24814888

[24] Scotti L, Abramovich D, Pascuali N, Durand LH, Irusta G, de Zuniga I, et al Inhibition of angiopoietin-1 (ANGPT1) affects vascular integrity in ovarian hyperstimulation syndrome (OHSS). Reprod Fertil Dev. 2014. 10.1071/RD13356 25485810

[25] Hua J, He ZG, Qian DH, Lin SP, Gong J, Meng HB, et al Angiopoietin-1 gene-modified human mesenchymal stem cells promote angiogenesis and reduce acute pancreatitis in rats. Int J Clin Exp Pathol. 2014; 7(7):3580–95. 25120736 PMC4128971

[26] Xue G, Yan HL, Zhang Y, Hao LQ, Zhu XT, Mei Q, et al c-Myc-mediated repression of miR-15-16 in hypoxia is induced by increased HIF-2alpha and promotes tumor angiogenesis and metastasis by upregulating FGF2. Oncogene. 2015; 34(11):1393–406. 10.1038/onc.2014.82 24704828

[27] Brown KC, Lau JK, Dom AM, Witte TR, Luo H, Crabtree CM, et al MG624, an alpha7-nAChR antagonist, inhibits angiogenesis via the Egr-1/FGF2 pathway. Angiogenesis. 2012; 15(1):99–114. 10.1007/s10456-011-9246-9 22198237

[28] Han H, Cao FL, Wang BZ, Mu XR, Li GY, Wang XW. Expression of angiogenesis regulatory proteins and epithelial-mesenchymal transition factors in platelets of the breast cancer patients. ScientificWorldJournal. 2014; 2014:878209 10.1155/2014/878209 25379550 PMC4212629

[29] Zhu K, Pan Q, Zhang X, Kong LQ, Fan J, Dai Z, et al MiR-146a enhances angiogenic activity of endothelial cells in hepatocellular carcinoma by promoting PDGFRA expression. Carcinogenesis. 2013; 34(9):2071–9. 10.1093/carcin/bgt160 23671131

[30] Lee MC, Lopez-Diaz FJ, Khan SY, Tariq MA, Dayn Y, Vaske CJ, et al Single-cell analyses of transcriptional heterogeneity during drug tolerance transition in cancer cells by RNA sequencing. Proc Natl Acad Sci U S A. 2014; 111(44):E4726–35. 10.1073/pnas.1404656111 25339441 PMC4226127

[31] Pollen AA, Nowakowski TJ, Shuga J, Wang X, Leyrat AA, Lui JH, et al Low-coverage single-cell mRNA sequencing reveals cellular heterogeneity and activated signaling pathways in developing cerebral cortex. Nat Biotechnol. 2014; 32(10):1053–8. 10.1038/nbt.2967 25086649 PMC4191988

