# Correction: RNA-Seq Reveals the Angiogenesis Diversity between the Fetal and Adults Bone Mesenchyme Stem Cell

**DOI:** 10.1371/journal.pone.0159221

**Published:** 2016-07-08

**Authors:** Xin Zhao, Yingmin Han, Yu Liang, Chao Nie, Jian Wang

There is an error in affiliation 2 for authors Xin Zhao, Chao Nie, and Jian Wang. Affiliation 2 should be: BGI-Shenzhen, Shenzhen, 518083, China.
