# Supplementary figures and images for: RNA-Seq Reveals the Angiogenesis Diversity between the Fetal and Adults Bone Mesenchyme Stem Cell
Source: PLoS One. 2016 Feb 22;11(2):e0149171. doi: 10.1371/journal.pone.0149171 (PMC4764296; doi:10.1371/journal.pone.0149171)

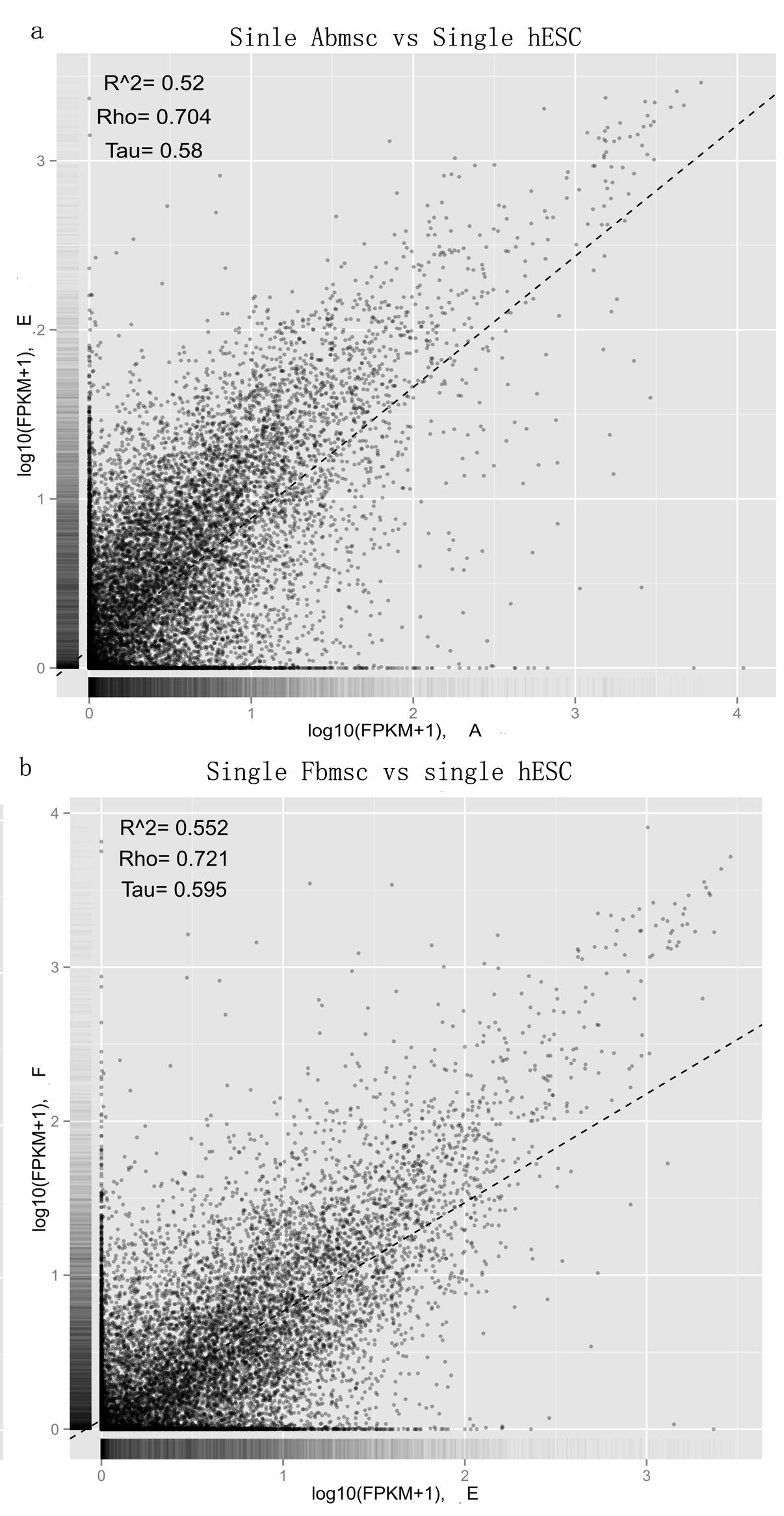

Supplement: S1 Fig — a) single Abmsc vs single hESC; b)single Fbmsc vs single hESC. All RefSeq genes expressed in at least one of the samples with FPKM ≥ 1 were used for the analysis. (TIF) [file pone.0149171.s001.tif]

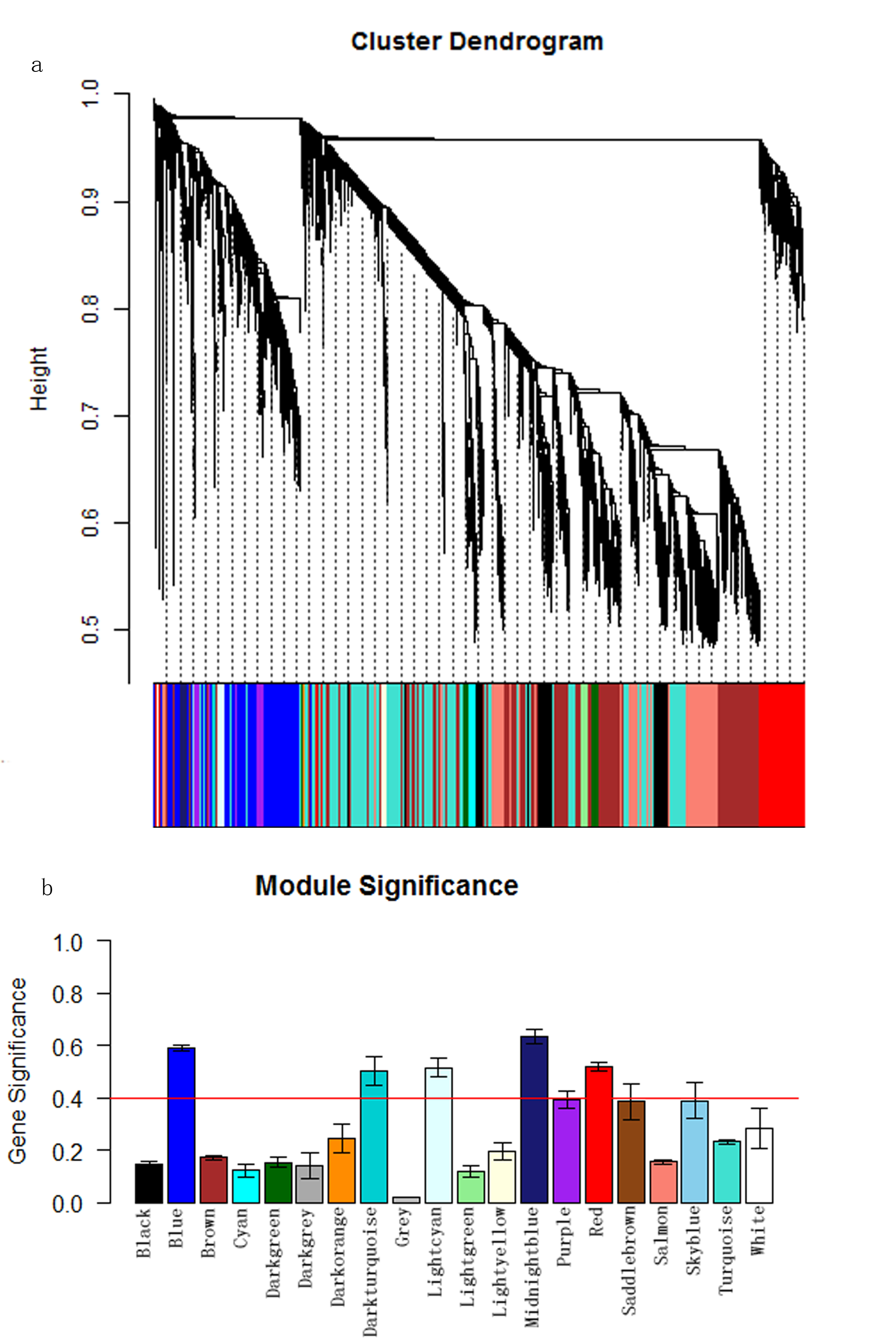

Supplement: S2 Fig — The picked module was matched two requirements: a) the cluster with the minim gene cluster of 10 and b) with the higher gene significance of 0.4. Other modules were abolished. (TIF) [file pone.0149171.s002.tif]

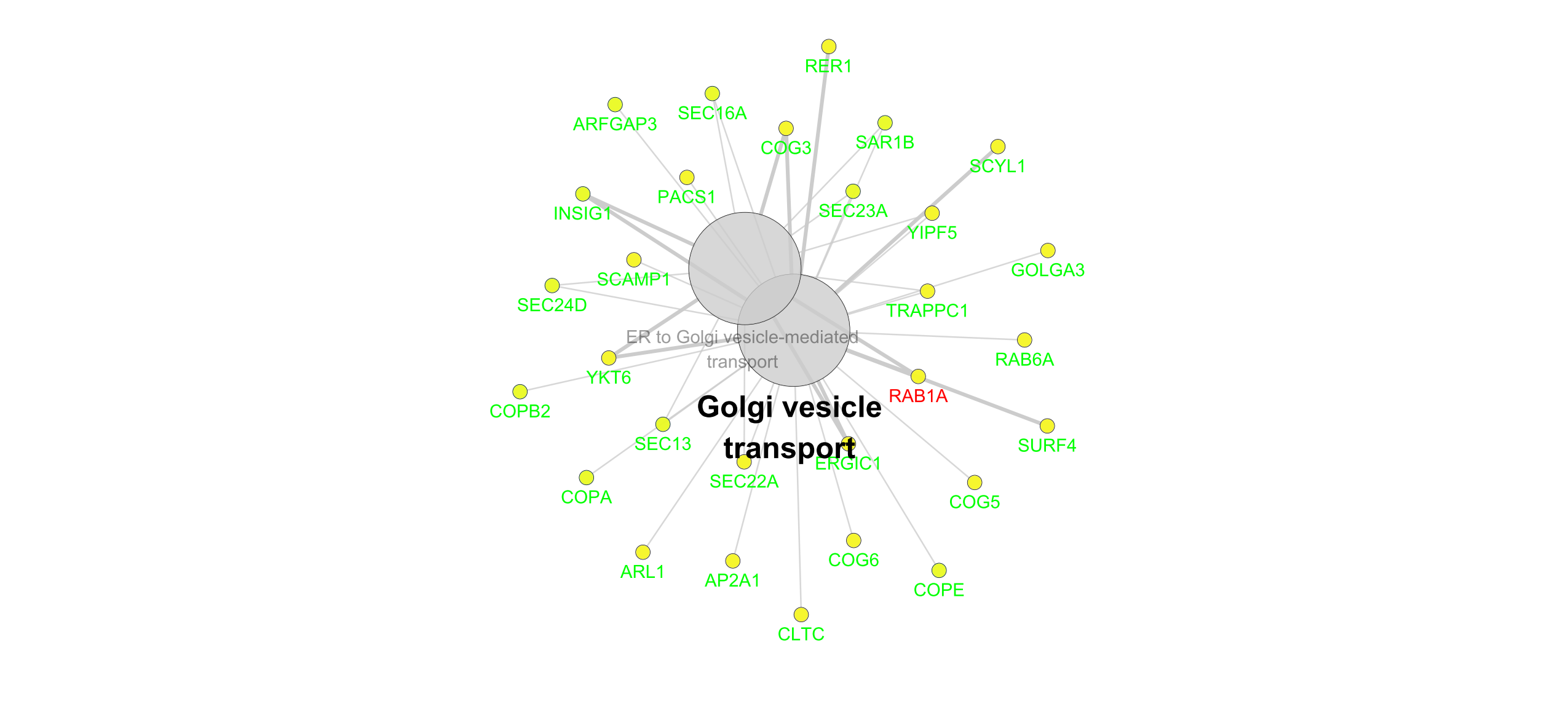

Supplement: S3 Fig — Before each viewing modules, parameters of P<10−5, %associated gene >8, and kappa = 0.5 were used to decrease the genes and Go terms in the module. The colors in the network showed the different cell lines expression. Red mean Abmsc showed the highest expression among the cell lines; while green mean Fbmsc and purple mean hESC. (TIF) [file pone.0149171.s003.tif]
