# Supplementary material for: RNA-Seq Reveals the Angiogenesis Diversity between the Fetal and Adults Bone Mesenchyme Stem Cell
Source: PLoS One. 2016 Feb 22;11(2):e0149171. doi: 10.1371/journal.pone.0149171 (PMC4764296; doi:10.1371/journal.pone.0149171)
Supplement: S1 Table — (DOCX) [file pone.0149171.s005.docx]

S1 Table. The primer of the TaqMan

| Gene Name | | Sequence |
| --- | --- | --- |
| RPLP0 | -F | 5’-GAGACGGATTACACCTTC -3’ |
|  | -R | 5’-AGACCAAATCCCATATCC -3’ |
|  | -P | 5’-(FAM) CCTCCGACTCTTCCTTGGCTT (BHQ1)-3’ |
| ACTB | -F | 5’-CATCCTCACCCTGAAGTA -3’ |
|  | -R | 5’-AGGTCTCAAACATGATCTG -3’ |
|  | -P | 5’-(FAM) ACGGCATCGTCACCAACTGG (BHQ1)-3’ |
| B2M | -F | 5’- CCTGAATTGCTATGTGTC -3’ |
|  | -R | 5’- CAGTGTAGTACAAGAGATAGA -3’ |
|  | -P | 5’-(FAM) CATCCATCCGACATTGAAGTTGACT (BHQ1)-3’ |
| RPS18 | -F | 5’- CGAAGATATGCTCATGTG -3’ |
|  | -R | 5’- GTGGATTCTGCATAATGG -3’ |
|  | -P | 5’-(FAM) AGCAGACATTGACCTCACCAAGA (BHQ1)-3’ |
| YWHAZ | - F | 5’- CGCTAATAATGCAATTACTG -3’ |
|  | - R | 5’-ACCTGTCATAAATCGTAAAC-3’ |
|  | - P | 5’-(FAM) CTTGACATTGTGGACATCGGATACC (BHQ1) -3’ |
| GAPDH | - F | 5’- GGACCTGACCTGCCGTCTAG -3’ |
|  | - R | 5’- TAGCCCAGGATGCCCTTGAG -3’ |
|  | -P | 5’-(FAM) CCTCCGACGCCTGCTTCACCACCT (BHQ1)-3’ |
