# Supplementary material for: RNA-Seq Reveals the Angiogenesis Diversity between the Fetal and Adults Bone Mesenchyme Stem Cell
Source: PLoS One. 2016 Feb 22;11(2):e0149171. doi: 10.1371/journal.pone.0149171 (PMC4764296; doi:10.1371/journal.pone.0149171)
Supplement: S2 Table — (DOCX) [file pone.0149171.s006.docx]

S2 Table. Basic quality of RNA-seq

| Sample ID | Total reads | Q20% | Q30% | GC% | Error% |
| --- | --- | --- | --- | --- | --- |
| A002 | 63,087,098 | 98.05-96.40 | 93.9-91.26 | 44.53-43.50 | 0.01-0.01 |
| A003 | 65,999,024 | 97.83-96.04 | 93.55-90.67 | 44.16-43.10 | 0.01-0.01 |
| A005 | 60,645,516 | 97.78-96.19 | 93.52-91.17 | 43.6-42.44 | 0.01-0.01 |
| A006 | 74,989,318 | 97.71-96.21 | 93.48-90.99 | 44.03-43.12 | 0.01-0.01 |
| A008 | 9,746,616 | 97.08-95.89 | 92.07-90.66 | 43.99-43.27 | 0.01-0.01 |
| A009 | 10,710,478 | 97.27-96.03 | 92.44-90.89 | 43.29-42.79 | 0.01-0.01 |
| A010 | 8,508,106 | 97.86-96.85 | 93.82-92.50 | 44.49-43.81 | 0.01-0.01 |
| A017 | 6,947,732 | 98.21-97.57 | 95.44-94.84 | 42.27-42.42 | 0.01-0.01 |
| F001 | 59,351,984 | 98.18-97.51 | 94.98-94.03 | 43.97-43.51 | 0.01-0.01 |
| F002 | 8,030,908 | 97.96-97.40 | 95.29-94.93 | 44.93-44.46 | 0.01-0.01 |
| F003 | 75,121,958 | 98.46-97.55 | 95.41-93.92 | 45.49-44.64 | 0.01-0.01 |
| F004 | 13,156,610 | 97.02-97.57 | 91.6-88.6 | 44.5-43.4 | 0.01-0.01 |
| F005 | 6,433,432 | 98.23-97.75 | 95.4-95.23 | 44.65-43.67 | 0.01-0.01 |
| F006 | 6,249,128 | 98.17-97.58 | 95.31-94.88 | 43.96-43.28 | 0.01-0.01 |
| F007 | 7,872,906 | 98.35-97.88 | 95.59-95.45 | 44.18-43.66 | 0.01-0.01 |
| F008 | 10,281,728 | 97.52-96.25 | 93.01-91.25 | 44.21-43.61 | 0.01-0.01 |
| F009 | 7,234,340 | 98.37-97.98 | 95.76-95.73 | 43.9-43.27 | 0.01-0.01 |
| F010 | 6,622,286 | 98.17-97.62 | 95.46-95.00 | 43.25-43.34 | 0.01-0.01 |
| F011 | 9,025,436 | 97.59-96.35 | 93.17-91.16 | 45.25-44.23 | 0.01-0.01 |
| F012 | 6,920,288 | 98.29-97.78 | 95.53-95.26 | 44.1-43.5 | 0.1-0.1 |
| F013 | 61,723,926 | 97.21-95.65 | 92.50-90.31 | 44.31-42.87 | 0.01-0.02 |
| F014 | 56,502,374 | 97.41-95.37 | 92.9-89.59 | 45.53-43.91 | 0.01-0.02 |
| F015 | 7,140,262 | 96.11-94.68 | 90.28-88.49 | 44.5-43.3 | 0.01-0.01 |
| A100(pool) | 53,944,088 | 98.03-97.13 | 94.58-93.31 | 45.45-44.43 | 0.01-0.01 |
| F100(pool) | 62,823,250 | 97.26-95.58 | 92.63-89.89 | 44.44-43.28 | 0.01-0.02 |

The results from the table were from read1 and read2
