# Supplementary material for: RNA-Seq Reveals the Angiogenesis Diversity between the Fetal and Adults Bone Mesenchyme Stem Cell
Source: PLoS One. 2016 Feb 22;11(2):e0149171. doi: 10.1371/journal.pone.0149171 (PMC4764296; doi:10.1371/journal.pone.0149171)
Supplement: S3 Table — (DOCX) [file pone.0149171.s007.docx]

S3 Table. The P value of the module in the network

| Module | Gene numbers | P |
| --- | --- | --- |
| darkgrey | 23 | 0.859023 |
| lightgreen | 47 | 0.550082 |
| cyan | 64 | 0.664146 |
| darkgreen | 128 | 0.58132 |
| salmon | 627 | 0.501175 |
| black | 320 | 0.536799 |
| brown | 1151 | 0.484428 |
| saddlebrown | 14 | 0.017012^*^ |
| skyblue | 15 | 0.016707^*^ |
| lightcyan | 55 | 0.000258^**^ |
| darkturquoise | 29 | 0.000724^**^ |
| midnightblue | 58 | 1.55×10^-6**^ |
| blue | 638 | 2.25×0^-7**^ |
| purple | 108 | 0.005908^**^ |
| lightyellow | 47 | 0.294196 |
| white | 17 | 0.069054 |
| darkorange | 20 | 0.142239 |
| red | 382 | 1.19×10^-5**^ |
| turquoise | 1204 | 0.511302 |
| grey | 1 | 0.929576 |

P*<0.05, P**<0.01
